# Veterinary Pharmaceutical Safety Competencies for New Graduates: Description of an Interprofessional Simulated Learning Activity

**DOI:** 10.1111/jvp.70091

**Published:** 2026-06-23

**Authors:** Margene Anderson, Carrie Schroeder, Kristopher M. Schroeder

**Affiliations:** ^1^ School of Veterinary Medicine University of Wisconsin Madison Wisconsin USA; ^2^ School of Medicine and Public Health University of Wisconsin Madison Wisconsin USA

**Keywords:** competency based veterinary education, experiential learning, interprofessional education, medication safety, prescribing practices, simulation training, veterinary drug diversion, veterinary pharmaceutical prescribing safety, veterinary pharmaceutical risk mitigation training, veterinary pharmacology

## Abstract

The misuse and abuse of veterinary anesthetic and analgesic agents pose significant public health risks, particularly with the growing presence of ultrapotent opioids and other anesthetics in the illicit drug trade. In response, there is increasing emphasis on incorporating medication safety training into veterinary curricula. This paper has two main objectives: first, to describe an evidence‐based, interprofessional simulation activity designed to raise awareness about the diversion and misuse of veterinary opioids and alpha‐2 agonists, while also developing basic life support skills for responding to accidental exposure; second, to propose three veterinary medication safety education sub‐competencies aligned with CBVE Domain 4 and proposed discipline‐level pharmacology competencies. These are aimed at equipping veterinary students to mitigate human health risks arising from exposure to veterinary medications, especially ultrapotent opioids and alpha‐2 agonists.

## 1 Introduction

The misuse and abuse of veterinary anesthetic and analgesic agents pose significant public health risks, particularly due to the growing presence of ultrapotent opioids, alpha‐2 agonists, and other veterinary medications in the illicit drug trade. In response, multiple publications have called for greater emphasis on medication safety education within veterinary curricula (Aarnes et al. 2022; Mason et al. 2018; Mitek et al. 2023; Chaney et al. 2024).

This paper has two primary aims. First, it describes an interprofessional simulated learning activity as an evidence‐based approach to integrating veterinary opioid safety training into an advanced anesthesia clerkship. Second, it proposes three sub‐competencies aligned with the Competency‐Based Veterinary Education (CBVE) framework, an American Association of Veterinary Medical Colleges (AAVMC) initiative for veterinary undergraduate and Doctor of Veterinary Medicine (DVM) programs, specifically Competency 4.2: “Promotes the health and safety of people and the environment” (Jawa et al. 2024, 9), with a focus on mitigating risks to human health from accidental or illicit exposure to veterinary medications.

## 2 The Risk of Harm to Humans From Exposure to Veterinary Medicines

Although accidental exposure to potent veterinary tranquilizers and anesthetic agents poses minimal risk to veterinary professionals (Haymerle et al. 2011; Woodward 2009), the growing misuse and abuse of these powerful—and often poorly understood—agents is of increasing concern to both human and veterinary healthcare professionals (Aarnes et al. 2022; Mason et al. 2018; Mitek et al. 2023; Chaney et al. 2024). Drug diversion and human misuse of veterinary medications are not a new phenomenon. The illicit use of the veterinary anesthetic phencyclidine (PCP) peaked in the 1970s, while ketamine misuse surged throughout the 1980s and 1990s, ultimately leading to the classification of both as Schedule III controlled substances in 1999 (Brenner 2024; Bischoff 2012).

More recently, the emergence of ultra‐potent synthetic opioids has dramatically worsened the opioid crisis in the United States (Mason et al. 2018; United States Drug Enforcement Administration (USDEA), n.d.; Mason et al. 2018; Weiman 2024; National Institute on Drug Abuse, n.d.; Drug Enforcement Administration 2019; Quail 2022). These opioids, including fentanyl and carfentanil, carry a high risk of inducing severe and often fatal respiratory depression (Eubanks et al. 2023; Zawilska et al. 2021). Ultra‐potent synthetic opioids are now implicated in the majority of overdose deaths, with mortality rates increasing by 750% between 2015 and 2022 (National Institute on Drug Abuse, n.d.; Drug Enforcement Administration 2019; Zawilska et al. 2021).

The use of veterinary drugs as adulterants in synthetic opioids and other illicit substances is rising sharply, with Alpha‐2 adrenergic receptor agonists—such as xylazine and medetomidine—emerging as particularly concerning threats (Pergolizzi et al. 2023; Friedman et al. 2022; Sugarman et al. 2024). In May 2024, the Center for Forensic Science Research and Education issued a “toxic adulterant alert” following a surge in overdose outbreaks linked to opioids and cocaine adulterated with medetomidine (Center for Forensic Science Research and Education 2024). Municipalities including Madison, Wisconsin, and Chicago subsequently released their own public health overdose alerts (Public Health Madison and Dane County 2024; Chi Health Alert Network 2024).

Alpha‐2 agonists pose serious risks to human health, including the development of disfiguring wounds and enhanced sedation that cannot be reversed with naloxone, compounding the dangers of synthetic opioids (Alexander et al. 2022). While there are other adulterants found in the illicit drug supply, this work focuses on the most frequently diverted and harmful veterinary medications—specifically synthetic opioids and alpha‐2 agonists. Although not an alpha‐2 agonist, the anthelmintic drug levamisole is another common adulterant found in illicit cocaine (Lee et al. 2012; Midthun et al. 2021). One report indicated levamisole contamination in 79% of the illicit cocaine supply, at times comprising up to 74% of a given sample by weight (Lee et al. 2012). Misuse of levamisole has been associated with severe adverse effects, including leukopenia and agranulocytosis (Midthun et al. 2021).

The American Veterinary Medical Association (AVMA) has indicated that drugs labeled for veterinary use are entering the illicit drug supply on a large scale through illegal importation rather than diversion from legitimate veterinary medicine channels (American Veterinary Medical Association 2023). However, veterinary practices are often targeted by individuals with intentions to divert medications prescribed for their animals for personal use (Anand and Hosanager 2021; Sharma et al. 2024). Tramadol, buprenorphine, gabapentin, and trazodone represent prescribed medications thought to be frequently diverted by pet owners for personal use (Sharma et al. 2024). These medications are often stocked in clinics and distributed directly to a client, which can leave veterinary clinics vulnerable to clients who engage in ‘vet shopping,’ exploiting their pet to obtain medications (Sharma et al. 2024; Kao‐Ping et al. 2022; Redding 2023).

## 3 Veterinary Opioid Safety Training: An Interprofessional Simulated Learning Activity

Due to the risk of harmful human exposure to veterinary medications—especially synthetic opioids and Alpha 2 agonists—veterinary training should equip day one ready graduates to identify diversion attempts as well respond effectively to acute exposure incidents. Following is a description of an evidence‐based learning intervention veterinary education programs might consider employing as an element of comprehensive veterinary medication safety training:

Annually, twelve to sixteen 4th‐year Doctor of Veterinary Medicine (DVM) students enroll in the Exotic Animal Anesthesia elective, an advanced anesthesia rotation at the University of Wisconsin School of Veterinary Medicine. The rotation is supervised by two board‐certified veterinary senior faculty anesthesiologists in the School of Veterinary Medicine. A core learning experience in this rotation is centered around a half‐day training exercise at the UW Health Clinical Simulation Center, located at the UW Health (human) Hospital on the University of Wisconsin—Madison Campus. The experience includes four components: (1) Interactive lecture; (2) High fidelity simulation responding to an accidental opioid overdose; (3) Abridged introduction to human advanced life support and human airway management; (4) Full group debrief and reflection.

The learning experience was designed to be intentionally interprofessional, co‐designed by faculty from the Department of Anesthesiology at the University of the Wisconsin School of Veterinary Medicine (SVM), faculty from the Department of Anesthesiology at the Wisconsin School of Medicine and Public Health (SMPH), and certified staff from the Clinical Simulation Program at the SMPH. Faculty from both the human and animal hospital anesthesia departments supervised the activities. Residents from the human anesthesia department were involved in demonstrations and actively participated in the didactic interactive lecture, full group debrief, and reflection portions of the experience.

The session began with a 60 min interactive lecture that included all faculty, student, and resident participants. The interactive lecture was led by a senior boarded human clinical anesthesiologist faculty, who systematically introduced a variety of topics relevant to human exposure to veterinary anesthetics: veterinary anesthetic pharmacology/pharmacokinetics, exposure mechanism/diagnosis, emergency response and basic life support, and an introduction to advanced life support. The veterinary students had background knowledge of veterinary anesthetic pharmacology/pharmacokinetics and airway and hemodynamic maintenance in animal patients, but no prior knowledge of or preparation for responding to an adverse human exposure to veterinary anesthetics was expected prior to the experience.

After the interactive lecture, veterinary students were divided into equal groups of four and rotated through three stations: High‐fidelity simulation; abridged introduction to human advanced life support; and human airway management technical skill practice. The simulation followed a standard simulation sequence, beginning with a pre‐brief to set the rules of engagement with the scenario, emphasizing suspension of disbelief. Additionally, the veterinary students were reminded that any performance in the simulation center would not negatively impact participants following the simulation encounter. During the pre‐brief, the students were also introduced to a high‐fidelity ‘true‐to‐life’ manikin outside of the simulation context. The manikins used in these scenarios are unique in that they feature the ability to simulate several physiologic responses that might be encountered in the treatment of veterinary anesthetic exposure. For example, pupil size can be manipulated to demonstrate opioid effects, breath sounds can be changed to demonstrate hypopnea related to opioid effects, and peripheral pulses can be manipulated to demonstrate hypotension that might be found in arrest scenarios. Students had an opportunity before the scenario to evaluate these manikin features and note what represented normal physical examination findings.

When the simulation began, students were presented with a scenario where a veterinary technician calls for help, reporting that their colleague in the adjoining room had been preparing an immobilizing dose of anesthetic for an upcoming large animal anesthetic procedure and now required medical assistance. Upon entering the room, the students found their simulated colleague on the floor, although still responsive and able to answer questions, albeit slowly and in a confused fashion (question answering is facilitated via a speaker in the manikin that is controlled in an adjacent room behind obscured glass). Initially, the simulated colleague had normal hemodynamics but quickly decompensated to a state where they were unconscious, apneic, and hypotensive.

Ideally, participants would recognize from the initial prompting (including a nearby syringe labeled as containing carfentanil) and state of their colleague that a veterinary anesthetic exposure had occurred. The objective was for the students to apply the earlier didactic teaching and respond systematically: Call emergency service; administer reversal agents; evaluate the patient for the presence of respiratory arrest and cardiovascular collapse; begin compressions; initiate airway support via bag‐mask ventilation while preparing for more invasive airway maneuvers; attach an automated external defibrillator (AED); obtain intravenous access; and treat hemodynamic compromise.

Following the simulation experience, learners debriefed with the faculty who conducted the scenario. Debrief is always the critical last step in any well‐designed simulated learning event; learners reflect on what went well, where struggles were encountered, and identify opportunities for improvement in knowledge and procedural skill (Redding 2023). The debrief is also an opportunity to consolidate takeaway points. In this incident key takeaway points included (1) identifying features of a potential adverse response to opioid exposure; (2) calling for help early in an arrest scenario improves outcomes; (3) bag‐mask ventilation represents an important airway management skill. This technique should be considered in situations where more advanced airway management may be challenging; (4) in the context of arrest following ultrapotent opioid exposure, consider increasing opioid receptor antagonist dosing, be prepared for renarcotization, and repeat opioid receptor antagonists as necessary; (5) consider the dangers associated with ultrapotent opioid exposure and ensure that future use is associated with the use of appropriate personal protective equipment (PPE) and handling/exposure protocols.

The second station was basic human airway management. Veterinary students, facilitated by human anesthesiology residents, practiced hands‐on training with high fidelity human airway management trainers. Specific attention was directed at optimizing bag‐mask ventilation as this is recognized as a skill that can benefit greatly from appropriate technique utilization. In addition, learners practiced oral and nasal airway sizing and insertion technique and experienced how these devices enhanced bag‐mask ventilation.

At the third station, learners were introduced to an abridged version of advanced life support airway management. Learners practiced supraglottic airway device insertion with a selection of first and second generation supraglottic airway devices. Finally, direct and video laryngoscopy technique and optimization strategies were repeated until learners gained procedural comfort. Human airway management technical skills training was facilitated by early‐career faculty human anesthesiologists, fellows, and residents. Instructors emphasized comparative anatomy over actual technique with the intention of piquing veterinary students' interest in potentially attaining human ACLS certification. The justification for this recommendation was that in a human clinical arrest scenario, a veterinarian may represent the most skilled medical professional present and certification would provide the appropriate skills set to respond to a human emergency.

Finally, all faculty, resident, and student participants regrouped for a whole group debriefing. The faculty approached this whole group debrief as a discussion rather than a didactic interactive lecture, encouraging participants to share prior knowledge and experiences. The focus of the discussion was the prominence of veterinary ultraopioid, alpha‐2 antagonists, and other veterinary medications as a threat to public health. However, the facilitators moved the conversation to concerns over medication diversion and misuse amongst health care workers, veterinary and human health professionals, and individual clients and patients. This aspect of the discussion included instruction on recognizing common abuse signs and behaviors veterinarians should be cognizant of and how to access support for individuals who may have an abuse disorder. Students were encouraged to ask questions and share their own concerns regarding mitigating exposure to opioids, and other commonly diverted veterinary medications, in practice.

## 4 Leveraging Evidence‐Based Instructional Practices

The American Association of Medical Colleges has issued five recommendations for effectively integrating safe opioid prescribing into clinician training in human health profession education. The learning experience described here incorporates three of the five: experiential learning strategies; developmentally appropriate and integrated content; inter‐professional educational opportunities (Mitek et al. 2023). The inclusion of a high‐fidelity simulation event exemplifies instructional methods that are anchored in the learning sciences, specifically leveraging the benefits of experiential learning and arousal theory.

Well‐designed simulation exercises like the simulation event detailed in the prior section are a prime example of experiential learning, which emphasizes the importance of learning through direct experience, reflection, and experimentation (Jefferies et al. 2024). Simulation events incorporate personal involvement, practical problem‐solving, and reflection, stimulating both a cognitive and affective response that creates a stronger foundation for memory encoding (Kolb 2014; Van Merrienboer and Kirschner 2018).

This specific simulation was built around an intense emergent event requiring time‐sensitive response and an increasingly heightened a sense of urgency, increasing the likelihood that students will experience emotional arousal. Emotional arousal significantly influences memory encoding due to an interplay between the hippocampus and the amygdala. When an experience evokes a strong emotional response, the amygdala becomes highly active, signaling to other brain regions to initiate the release of key neurotransmitters, including norepinephrine and glutamate, enhancing the encoding process in the hippocampus, leading to more deeply encoded and resilient memories, and thus strengthening recall (Ma et al. 2018; McGaugh 2018; Çetin et al. 2016).

A well‐designed simulation event ends with a faculty‐led debrief. As was done in this case, the debrief is meant for students to engage in self‐assessment focusing on clinical decision making and technical skill development (Jefferies et al. 2024; Van Merrienboer and Kirschner 2018). The debrief also plays a role in self‐monitoring affective responses to emotionally heightened experiences. While heightened emotions can redirect cognitive attention in a way that impedes learning (Hinojosa et al. 2009), the debrief session allows students to learn about their potential emotional response in emergent situations. This debrief also engaged students in a frank discussion about veterinary medicine misuse both within the general population and amongst veterinary professionals. The faculty discussed ‘warning signs’ that could indicate medication abuse including socially withdrawing, unaccounted for narcotics, doctor shopping and using their animal's health as an access point to divert veterinary medications for personal use.

Engaging in the simulation experience prior to discussing this topic seemed to make the conversation more meaningful, and thus impactful. Emotional arousal impacts memory encoding, as discussed above, but also instigates emotional priming, which can serve as an entry way into challenging discussion by inciting curiosity, empathy, or even a mild level of anxiety. This often results in enhanced activity engagement and cognitive growth (Hinojosa et al. 2009; Storbeck and Clore 2008). Anecdotally, students report being incredibly engaged throughout this encounter and acutely aware of what their potential role might be in exposure and arrest scenarios and how this training may be critical to the resuscitation of a future colleague or even a member of the public.

## 5 Aligning With Competency‐Based Veterinary Education and Veterinary Pharmacology Competencies

Veterinary students who participated in this learning intervention reported that it was the first time they had received instruction on veterinary drug diversion and misuse during their 4 years of veterinary education. While medical schools have increasingly recognized the need to address opioid misuse—with approximately 90% of U.S. human medical curricula now including opioid safety training (Mitek et al. 2023)—a similar emphasis has yet to be broadly implemented in veterinary programs.

Ultrapotent opioids and alpha‐2 agonists will remain essential components of veterinary anesthetic and analgesic protocols, particularly in large animal medicine, where they often represent the safest sedation options for both animal and practitioner (Veterinary Anesthesia and Analgesia, 6th Edition of Lumb & Jones). As such, newly graduated veterinarians—like their counterparts in human medicine—should be prepared to understand and mitigate the risks associated with veterinary drug diversion and human misuse (United States Drug Enforcement Administration (USDEA), n.d.; Kao‐Ping et al. 2022). This realization prompted the authors to reflect more broadly on the purpose and potential impact of the described learning intervention.

The educational activity outlined in this paper aligns with Competency 4.2 of the Competency‐Based Veterinary Education (CBVE) framework: “Promotes the health and safety of people and the environment.” (Chaney et al. 2024, 9). It presents a timely opportunity to expand the scope of veterinary medication safety education in alignment with evolving public health challenges.

Furthermore, there is a meaningful connection to the veterinary pharmacology discipline‐level competencies developed by Werners and Fajt in 2020 (Werners and Fajt 2023). Although these competencies, finalized through a two‐cycle Delphi process, were developed prior to the CBVE framework, the authors emphasize the alignment. The discipline‐level pharmacology competencies include Category 4: “Drug dispensing, regulations, and ethics,” which encompasses sub‐competencies such as:

1. Describing the advantages and disadvantages of standardized protocols for drug decision‐making:
2. Explaining the role of professional organizations and regulatory bodies in governing veterinary drug use; and
3. Understanding the importance of dispensing drugs in childproof containers

While these competencies provide an essential foundation, they do not explicitly address the veterinarian's role in mitigating harmful human exposure to veterinary medications. Nonetheless, they offer a valuable model for how safety education can be further integrated into veterinary curricula. In this context, the authors of this paper propose three new sub‐competencies for consideration by veterinary educators—designed to align with CBVE Competency 4.2 and the ethos of Category 4 in the Werners and Fajt framework. These proposed sub‐competencies were developed based on the learning objectives of the simulation‐based intervention described in this paper, informed by relevant literature on veterinary medication safety and public health risks, and structured to complement existing pharmacology competencies.

1. Identify public health and legal implications of the rising presence of veterinary medications, and specifically ultra‐potent opioids and alpha‐2 agonists, in the illicit drug supply:
2. Recognize risk factors that may lead to improper use and diversion of veterinary medications;
3. Practice basic life support skills for responding to human drug overdose due to accidental or intentional exposure to veterinary anesthesia and analgesia medications.

The authors acknowledge that effectively addressing veterinary medication safety—including the integration of the proposed sub‐competencies—will require a more comprehensive, curriculum‐wide approach rather than reliance on a single learning intervention. However, we believe that sharing this novel learning experience offers a valuable example of an evidence‐based instructional approach that not only supports medication safety education but also meaningfully contributes to the development and contextualization of the proposed sub‐competencies. This intervention illustrates how targeted educational strategies can inform broader curricular goals and help prepare veterinary graduates to address emerging public health risks.

## 6 Limitations

Implementing an interprofessional, simulation‐based learning intervention does require a collection of motivated faculty and dedicated simulation center staff. The initial creation of the simulation required approximately 2 h of intermittent discussions with simulation center staff. Of note, the faculty and staff who co‐designed the simulation had established working relationships that may have increased efficiency.

This experience does also require the ongoing dedication of multiple members of the Department of Anesthesiology to successfully demonstrate airway management and associated procedural skills. An inevitable challenge associated with this type of intervention is the scalability required to expand it to a full cohort of students, given the resource‐intensive nature of high‐fidelity simulation, the need for interprofessional collaboration, and the limited availability of simulation facilities and trained personnel.

One recurring topic during this program is the potential liability associated with providing emergency medical care. From a legal standpoint, it is considered misconduct for veterinarians to provide medical care to humans, as they are licensed specifically for animal care. This was addressed during the full debrief. The human anesthesiologists noted that in situations such as the simulation they had just experienced, a veterinarian may be the most medically skilled individual available to deliver timely, life‐saving assistance. Students are encouraged to seek legal counsel in their state or country of practice to gain a full understanding of the applicable legal landscape, including potential protections under Good Samaritan laws. Educators implementing similar programs are similarly advised to seek legal counsel regarding liability considerations and to ensure students understand both the limitations and protections relevant to emergency intervention.

The training that the students receive in this educational experience is not intended to serve as a replacement for formal Advanced Cardiovascular Life Support (ACLS) training. The aim is to raise awareness of the value of ACLS rather than to assess students' skills or knowledge acquisition. Course instructors emphasize the importance of viewing themselves as part of the broader medical and health professional community and encourage students to consider pursuing ACLS certification as part of their continued professional development.

The authors recognize that there are a number of Doctor of Veterinary Medicine programs that are not associated with a veterinary teaching hospital, nor do they have access to a human hospital with an advanced simulation center. There may be other interprofessional partnership veterinary education programs that could leverage, even if high fidelity simulation isn't possible. Regional technical schools and community‐based first responder training programs may be able to provide Basic Life Support, including responding to potential opioid overdose training that could be coupled with guided discussion.

## 7 Further Study

There are a number of potential formal areas of study that could arise from this pedagogically focused paper. Following the Werners and Fajt model, a formal—and even interprofessional—study to evaluate the necessity and validity of the proposed sub‐competencies is an area the authors plan to pursue. Another future potential avenue of study would be to assess students' knowledge acquisition and retention of the medical safety content and BCLS skills after participating in the described learning intervention, including the rate at which students pursued more formalized ACLS training.

## 8 Conclusion

Addressing human exposure to veterinary medications is a critical gap in veterinary education, especially given the significant role these drugs play in public health challenges such as opioid misuse and veterinary drug diversion. Veterinarians, with their unparalleled expertise in the pharmacology of potent anesthetics, should explore opportunities to collaborate with human healthcare educators to develop interprofessional medical safety education initiatives. A “day‐one‐ready” veterinarian must recognize their responsibility in shaping policy and preventing drug diversion, as this is a key component of public health.

The interprofessional learning experience described, along with the proposed sub‐competencies, could equip future veterinarians with the knowledge and skills needed to mitigate the risks associated with veterinary pharmaceuticals, including managing acute human exposures. The training exercise outlined here underscores the value of experiential learning in preparing veterinary students to address human health risks tied to their field. Through such innovative approaches, veterinary professionals can play an essential role in safeguarding both animal and human health, positioning them as key partners in combating medication misuse and promoting public safety.

## Author Contributions

Dr. Anderson was the primary author of the manuscript. Dr. K.M. Schroeder and Dr. C. Schroeder conceptualized and supervised the described intervention, and reviewed the manuscript for medical accuracy. All authors reviewed the manuscript for intellectual content, approved the final version to be published, and agree to be accountable for all aspects of the work in ensuring that questions related to the accuracy or integrity of any part of the work are appropriately investigated and resolved.

## Funding

The authors have nothing to report.

## Ethics Statement

The authors confirm that the ethical policies of the journal, as noted on the journal's author guidelines page, have been adhered to. No ethical approval was required as no animals were used.

## Conflicts of Interest

The authors declare no conflicts of interest.

## Data Availability

Data sharing is not applicable to this article as no new data were created or analyzed in this study.
